# Zika Virus Outbreak — Bangladesh, September–December 2024

**DOI:** 10.15585/mmwr.mm7501a1

**Published:** 2026-01-08

**Authors:** Jannatul Ferdous, Md Abdullah Omar Nasif, Gretchen Cowman, Immamul Muntasir, Mohammad Rashedul Hassan, Md Omar Qayum, Kyaw Thowai Prue Prince, Ahmed Nawsher Alam, Manjur Hossain Khan Jony, Fariha Masfiqua Malek, Rozina Afroz, Mahbubur Rahman, Tahmina Shirin

**Affiliations:** ^1^Field Epidemiology Training Program, Institute of Epidemiology, Disease Control and Research, Bangladesh; ^2^Institute of Epidemiology, Disease Control and Research, Bangladesh; ^3^Career Epidemiology Field Officer, Office of Readiness and Response, CDC.

**Summary**

**What is already known about this topic?**

In Bangladesh, Zika virus was first detected in 2014; subsequently, five cases of Zika virus disease were identified in 2023. Zika virus infection is often mild but rarely can result in neurologic complications, and, in early pregnancy, can cause severe congenital anomalies.

**What is added by this report?**

Ten Zika virus disease cases were detected in and around Dhaka, Bangladesh, during September–December 2024. Most cases were initially suspected to be dengue or chikungunya and were identified incidentally through a multiplex reverse transcription–polymerase chain reaction assay. No patients were pregnant, and none were hospitalized. Zika virus RNA was detected in local mosquitoes.

**What are the implications for public health practice?**

Integrated testing and surveillance for arboviral diseases might improve detection of Zika virus disease and support clinical management. Prevention through vector control and personal protection should also be emphasized.

## Abstract

Zika virus infection is transmitted to humans primarily through the bite of infected *Aedes* species mosquitoes. Although most Zika virus disease cases are mild or asymptomatic, severe neurologic complications can occur. Infection during pregnancy can result in severe congenital anomalies. In Bangladesh, Zika virus was first detected in an archived specimen from 2014; subsequently, five cases of Zika virus disease were identified in 2023. In September 2024, in response to identification of a confirmed Zika virus disease case in Bangladesh’s capital, Dhaka, in a woman aged 29 years who was initially thought to have dengue, the Institute of Epidemiology, Disease Control and Research (IEDCR) launched an outbreak investigation. After IEDCR notification to hospitals, five additional Zika virus disease cases were identified in four patients evaluated at three Dhaka hospitals and in a household contact of one of these patients. Another four Zika virus disease cases were identified through Zika virus testing of patients referred to IEDCR during a concurrent chikungunya outbreak. In total, 10 confirmed cases of Zika virus disease were detected in and around Dhaka during September–December 2024 in patients with no history of international travel. None of the patients was pregnant, and all recovered without hospitalization or complications. An entomological investigation detected Zika virus RNA in *Aedes* species mosquitoes in Dhaka. This investigation suggests sporadic Zika virus transmission occurs in Dhaka. Integrated testing and surveillance for arboviral diseases might improve detection of Zika virus disease and support clinical management in areas where transmission of multiple arboviral diseases occurs. Prevention of these infections through vector control and use of personal protective measures should also be emphasized. 

## Introduction

Zika virus is a single-stranded RNA virus belonging to the Flavivirideae family and was first identified in Uganda in 1947 (*1*). The first documented Zika virus disease outbreak occurred on the Pacific island of Yap in 2007 (*2*). An association between fetal microcephaly and congenital Zika virus infection was observed during a large outbreak in Brazil during 2015–2016 (*3*). Zika virus is primarily transmitted by the bite of infected *Aedes* species mosquitoes. Less common modes of transmission include intrauterine and intrapartum transmission, sexual transmission, transmission through breastfeeding, blood transfusion or laboratory transmission, and transmission through organ and tissue transplantation (*4*). Exposure to Zika virus in early pregnancy can result in congenital Zika syndrome, which is characterized by microcephaly, brain abnormalities, vision problems, low birth weight, and other conditions (*5*).

Zika virus was first detected in Bangladesh when reverse transcription–polymerase chain reaction (RT-PCR) testing of archived dengue-negative serum samples collected between 2013 and 2016 identified a single Zika-positive specimen from 2014 in a patient with no history of international travel (*6*). A study conducted by a nongovernmental research organization during July–December 2023 in the capital city of Dhaka identified five Zika virus disease cases among 152 febrile patients tested for dengue, Zika, and chikungunya viruses (*7*). No routine surveillance for Zika virus is conducted in Bangladesh, although it is a notifiable disease (*8*). In September 2024, the Institute of Epidemiology, Disease Control and Research (IEDCR), under the Ministry of Health and Family Welfare in Bangladesh, was notified by a private hospital in Dhaka of a laboratory-confirmed Zika virus disease case in a person with dengue-compatible symptoms and no history of international travel. This report describes the outbreak investigation that was prompted by identification of this case.

## Investigation and Results

### Identification of Index Case

On September 4, 2024, a woman in Dhaka aged 29 years who was not pregnant (patient A) and had symptoms of dengue (fever, joint pain, and rash) that began on August 31 received a positive Zika virus RT-PCR test result from a multiplex test (Genesig kit | Primer Design | United Kingdom) for dengue, chikungunya, and Zika virus while being evaluated at the outpatient department of hospital 1. The test had been requested because clinicians, after receipt of a negative rapid dengue nonstructural protein-1 (NS1) antigen diagnostic test result, suspected chikungunya. The multiplex test detected Zika virus RNA only. The hospital notified IEDCR and sent a serum sample to the IEDCR virology laboratory for confirmatory testing.* IEDCR also obtained a serum and urine sample from the patient. All samples tested positive for Zika virus RNA by RT-PCR. 

### Epidemiologic Investigation and Surveillance

On September 4, 2024, IEDCR initiated an investigation to identify the source of the Zika virus infection and determine whether additional cases were occurring. Because in Bangladesh the occurrence of single laboratory-confirmed case with suspected local transmission is treated as an outbreak and prompts an outbreak investigation and response, on September 9, after confirmation of the first case, IECDR declared a Zika virus disease outbreak in Dhaka. A confirmed case was defined as detection of Zika virus RNA by RT-PCR in serum or urine in the IEDCR laboratory. This activity was reviewed by IEDCR and CDC, deemed not research, and was conducted consistent with applicable federal law and CDC policy.^†^

After obtaining information about patient A’s clinical signs and symptoms from the treating physician at hospital 1, investigators visited the woman at home and collected information including demographic data, symptom onset date, clinical manifestations, and possible sources of exposure (e.g., mosquito bite, blood transfusion, organ transplantation, sexual contact, travel history, and travel history of sexual partners). As part of the investigation, all five of patient A’s asymptomatic household members (age range = 22–48 years) were interviewed about potential exposures and travel history, and all provided blood specimens. All five household contacts’ blood test results were negative for Zika virus. IEDCR reported the case to the International Health Regulations national focal point in Bangladesh, informed Dhaka hospitals and the public through a media briefing, and notified the Obstetrical and Gynecological Society of Bangladesh. IEDCR issued a statement to all government hospitals in Bangladesh instructing them to refer any patient with suspected Zika virus disease, chikungunya, or dengue for testing. 

After investigation of the index case, IEDCR established a suspected Zika virus disease case definition that consisted of onset of fever and maculopapular rash during the previous 2 weeks, with at least one of the following: conjunctivitis, arthralgia, or having a household member with confirmed Zika virus disease. In response to subsequent notifications of suspected Zika virus disease cases, IEDCR collected information from the reporting facilities, confirmed diagnoses by RT-PCR testing at the IEDCR virology laboratory, interviewed patients, and conducted active case finding at patients’ residences, including interviewing household members and conducting laboratory testing of those with suspected Zika virus disease.

### Identification of Cases by Hospitals

On September 17, IEDCR was notified by a second hospital in Dhaka (hospital 2) of two patients with RT-PCR–confirmed Zika virus disease (patients B and C) (Table). Both were evaluated as outpatients. Physicians had initially suspected dengue and ordered Zika virus testing after receiving negative results for dengue NS1 antigen. An investigation at patient B’s home identified a household member who met the suspected case definition and was later confirmed to have Zika virus disease (patient D). On October 5 and October 20, two additional hospitals (hospitals 3 and 4) in Dhaka reported laboratory-confirmed Zika virus disease cases in outpatients (patients E and F). No additional cases were identified among these patients’ household members.

**TABLE:** Characteristics of patients with laboratory-confirmed Zika virus disease cases — Bangladesh, September–December 2024

Patient	Age, yrs	Sex*	Date of symptom onset	Clinical features and other characteristics	Specimen positive for Zika virus RNA	Duration of illness, days	Residence
A^†^	29	Female	Aug 31	Fever, arthralgia, myalgia, rash, conjunctivitis, and headache	Serum, urine	8	Dhaka South
				Initially suspected to have chikungunya			
				Positive multiplex test result after negative rapid dengue antigen test result^§^			
B	38	Female	Sep 13	Fever, arthralgia, myalgia, rash, and headache	Urine	14	Dhaka North
				Confirmed by RT-PCR at hospital 2 after negative dengue test result			
C	23	Female	Sep 10	Fever, arthralgia, myalgia, rash, and headache	Serum	10	Dhaka North
				Confirmed by RT-PCR at hospital 2 after negative dengue test result			
D	36	Female	Sep 12	Fever, arthralgia, myalgia, rash, conjunctivitis, and headache	Serum, urine	7	Dhaka North
				Household contact of patient B			
E	42	Male	Sep 29	Fever, arthralgia, myalgia, and rash	Urine	7	Dhaka North
F	33	Female	Oct 18	Fever, arthralgia myalgia, and conjunctivitis	Urine	5	Dhaka North
G	44	Male	Nov 20	Fever, arthralgia, myalgia, rash, and conjunctivitis	Urine	6	Dhaka South
H	50	Male	Nov 23	Fever, arthralgia, myalgia, rash, conjunctivitis, and headache	Urine	6	Dhaka North
I	30	Female	Nov 25	Fever, arthralgia, myalgia, rash, conjunctivitis, and headache	Serum	8	Gazipur
J	52	Female	Dec 29	Fever, arthralgia, myalgia, rash, and headache	Urine	7	Dhaka South

**Abbreviation**: RT-PCR = reverse transcription–polymerase chain reaction.

* No patients with Zika were pregnant.

^†^ All five of patient A’s household members received negative test results.

^§^ The Zika outbreak was declared after diagnosis confirmation for patient A. Multiplex test: Primer Design dengue, zika and chikungunya virus multiplex kit.

### Identification of Cases Among Patients with Suspected Chikungunya Referred for Testing

During October, a concurrent chikungunya outbreak was detected in Dhaka (*9*). In response to IEDCR’s letter requesting referral of patients with suspected Zika virus disease, chikungunya, or dengue for testing, health facilities began referring patients with presumed chikungunya as part of this outbreak for testing. IEDCR established a sample collection booth and screened patients for testing using a questionnaire that collected demographic and clinical information. During October–December 2024, a total of 394 referred patients, most of whom were suspected to have chikungunya, provided serum samples for testing by RT-PCR multiplex assay. Among these, 34 (8.6%) met the suspected Zika virus disease case definition and provided urine samples for RT-PCR testing as well. Overall, four additional Zika virus disease cases were confirmed: three had Zika virus RNA detected in urine (patients G, H, and J) and one received a positive serum test result (patient I).^§^

### Characteristics of Persons with Zika Virus Disease 

Among the 10 patients with confirmed Zika virus disease cases identified during the outbreak, the median patient age was 37 years (range = 23–52 years); seven cases occurred in women, none of whom was pregnant (Table). All patients were interviewed using the same questionnaire. Sexual contact, blood transfusion, and organ transplantation were ruled out as possible routes of transmission. No patient had a history of international travel within the 2 weeks preceding symptom onset, and only two cases appeared to be epidemiologically linked. All patients had relatively mild illnesses, all received supportive care, and none were hospitalized. All patients had fever (≥100.4°F [≥38.0°C)]), arthralgias, and myalgias. Nine also had a generalized rash, seven experienced headaches, and six had conjunctivitis. The median duration of illness was 7 days (range = 5–14 days). Zika virus RNA was detected in the serum of two patients, the urine of six patients, and both the serum and urine of two patients. Nine patients lived in various locations in Dhaka, and one lived in the adjacent Gazipur district (Figure). Although there were concurrent dengue and chikungunya outbreaks, no co-infections were detected in patients with confirmed Zika virus disease.

**FIGURE:**
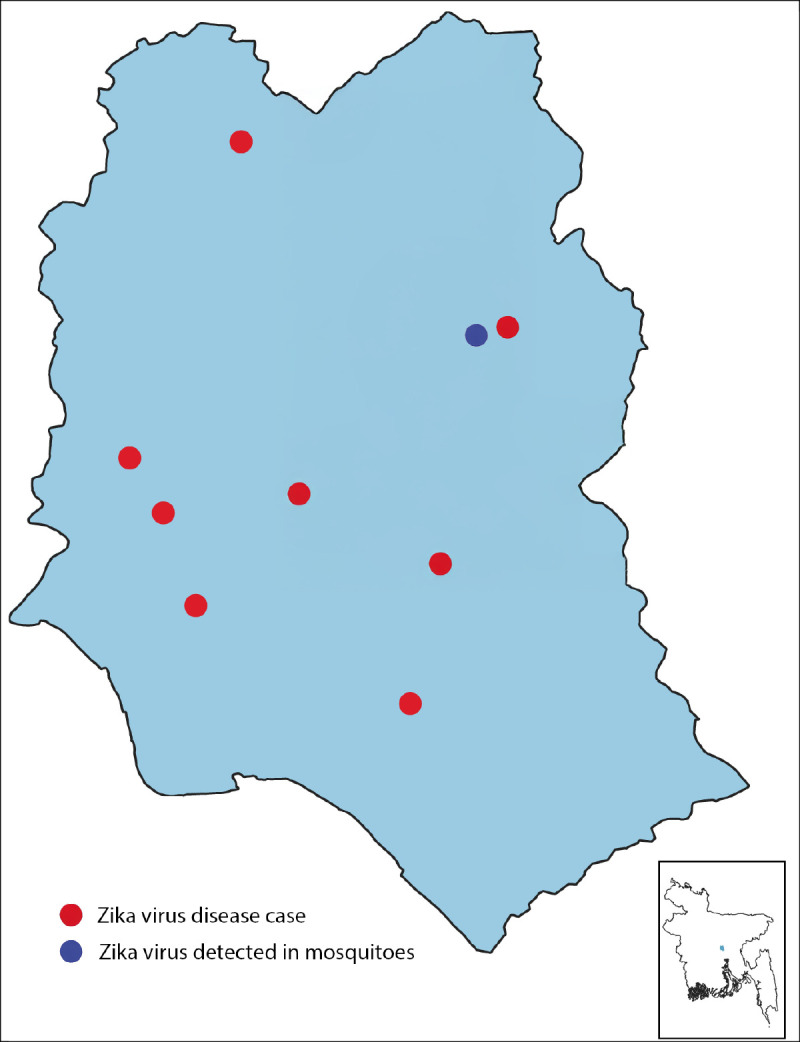
Distribution of Zika virus disease cases — Dhaka, Bangladesh, September–December 2024* * Of the 10 persons with confirmed cases, residence locations of nine are displayed. The tenth case occurred in the adjacent Gazipur district (north of Dhaka), which is not included on the map. Two patients lived in the same household.

### Entomological Investigation

To ascertain whether Zika virus was present in mosquitoes in Dhaka, IEDCR conducted an entomological investigation at seven sites. Each site was within approximately 0.5 miles (1 km) of the home of a patient with confirmed Zika virus disease.^¶^ Larvae were collected from one or two ponds or lakes at each site. The larvae were then combined by site, reared to adulthood in the entomology laboratory, and tested for Zika virus RNA by an RT-PCR multiplex test in the virology laboratory. Zika virus RNA was detected in a pooled sample of mosquitoes from one of the seven sites (Figure). 

## Public Health Response

The Public Health Emergency Operations Center of IEDCR coordinated the response, rapidly deploying teams to investigate each reported Zika virus disease case. IEDCR notified the media about the presence of Zika virus in Dhaka, and the information was distributed online to raise awareness among clinicians and the public. Zika virus screening was incorporated into evaluation of patients referred to IEDCR for RT-PCR testing for chikungunya. Screening for Zika virus, in addition to dengue and chikungunya, has continued during the 2025 dengue and chikungunya season (June–December), and surveillance for acute febrile illness has been initiated in six sites throughout Bangladesh where Zika has been designated a priority disease. In addition, IEDCR initiated an arboviral serosurvey in Dhaka to gain a better understanding of the presence and extent of Zika virus and other arboviruses in the city. Vector control programs for dengue, organized by city authorities are ongoing to reduce mosquito density. Although routine surveillance for Zika virus disease in obstetrics and gynecology departments of health care facilities has not been established, awareness has been raised that obstetric patients with clinical signs and symptoms compatible with Zika virus disease, dengue, or chikungunya should be referred to IEDCR for testing. Provisional data from 2025 indicate fewer than 10 confirmed Zika virus disease cases were identified in Bangladesh during June–November 2025 (IEDCR, unpublished data, November 2025). 

## Discussion

This investigation found that transmission of Zika virus is occurring in Dhaka, Bangladesh, and surrounding areas. This is the first reported Zika virus disease outbreak of its size in Dhaka, and 2024 is the second consecutive year that Zika virus disease has been detected in Dhaka. Although Zika virus was first identified in an archived sample from 2014, and the first confirmed outbreak occurred in 2023, reoccurrence in 2024 highlights the potential for the virus to become endemic in Bangladesh. The widespread geographic distribution of cases in 2024 suggests established circulation of infected mosquitoes in Dhaka; this distribution is distinctly different from that in 2023, when all five patients with confirmed Zika virus disease lived within a radius of approximately 0.5 miles (1 km) (*7*). The number of Zika virus disease cases detected likely does not reflect the true magnitude and geographic distribution of Zika virus transmission in Bangladesh. Cases are likely underreported because of the absence of systematic surveillance, limited availability of testing, occurrence of mild or asymptomatic infections, and possible misdiagnosis of Zika virus disease cases as chikungunya or dengue.

Dengue is an important public health concern in Bangladesh, with annual seasonal outbreaks (*10*). The existence of a national dengue surveillance system provides an opportunity to expand surveillance to include Zika virus disease and chikungunya, thereby enabling an integrated approach to monitoring and response to these mosquito-borne infections. The potential for severe neurologic complications of Zika virus disease, along with the risks during pregnancy, including congenital Zika syndrome, underscore the critical importance of detection of Zika virus disease in Bangladesh. These findings highlight the need to reduce transmission and prevent future outbreaks by improving vector control, using personal protection, ensuring timely diagnosis, and integrating Zika virus disease surveillance into the existing dengue surveillance system.

### Implications for Public Health Practice

Raising clinician awareness of the presence of Zika virus in Bangladesh might encourage routine Zika virus testing of febrile patients with suspected dengue or chikungunya. This is particularly important for pregnant women with febrile illness or rash to ensure early detection. Efforts to increase community awareness of the importance of preventing mosquito bites should be emphasized, especially for pregnant women.

Integration of Zika virus testing and surveillance with that for other arboviral diseases in patients with compatible signs and symptoms might improve the detection of Zika virus disease and support appropriate clinical management, including antenatal monitoring of pregnant patients with Zika virus disease. This outbreak suggests that Zika virus is established in mosquitoes in Bangladesh. Establishing Zika virus surveillance, strengthening vector control measures, and educating providers and the public are important to prevent further transmission, improve case detection, and guide clinical management.

## Footnotes

* Viral RNA was extracted from 140 *μ*L of serum using the QIAamp Viral RNA Mini Kit and eluted in 30 *μ*L of elution buffer. Multiplex real-time RT-PCR (targeting dengue, Zika, and chikungunya viruses) was performed on an ABI QuantStudio 5. Samples with cycle thresholds <50 and valid internal control were considered positive.

† 45 C.F.R. part 46, 21 C.F.R. part 56; 42 U.S.C. Sect. 241(d); 5 U.S.C. Sect. 552a; 44 U.S.C. Sect. 3501 et seq.

§ Among those tested, positive results for chikungunya, dengue, and Zika virus disease were received by 138, 11, and four persons, respectively.

¶ Nine confirmed Zika virus disease cases were identified in Dhaka. Seven sites were selected, covering the nine patients’ homes within a radius of approximately 0.5 miles (1 km). Two patients lived in the same household, and two patients lived within approximately 0.5 miles (1 km) of each other. No entomological investigation was carried out in Gazipur district.
